# Protein Linewidth and Solvent Dynamics in Frozen Solution NMR

**DOI:** 10.1371/journal.pone.0047242

**Published:** 2012-10-15

**Authors:** Ansgar B. Siemer, Kuo-Ying Huang, Ann E. McDermott

**Affiliations:** Department of Chemistry, Columbia University, New York, New York, United States of America; National Institute for Medical Research, Medical Research Council, United Kingdom

## Abstract

Solid-state NMR of proteins in frozen aqueous solution is a potentially powerful technique in structural biology, especially if it is combined with dynamic nuclear polarization signal enhancement strategies. One concern regarding NMR studies of frozen solution protein samples at low temperatures is that they may have poor linewidths, thus preventing high-resolution studies. To learn more about how the solvent shell composition and temperature affects the protein linewidth, we recorded ^1^H, ^2^H, and ^13^C spectra of ubiquitin in frozen water and frozen glycerol-water solutions at different temperatures. We found that the ^13^C protein linewidths generally increase with decreasing temperature. This line broadening was found to be inhomogeneous and independent of proton decoupling. In pure water, we observe an abrupt line broadening with the freezing of the bulk solvent, followed by continuous line broadening at lower temperatures. In frozen glycerol-water, we did not observe an abrupt line broadening and the NMR lines were generally narrower than for pure water at the same temperature. ^1^H and ^2^H measurements characterizing the dynamics of water that is in exchange with the protein showed that the ^13^C line broadening is relatively independent from the arrest of isotropic water motions.

## Introduction

Solid-state NMR on frozen protein solutions has a great potential for investigating protein folding, dynamical disorder, and soluble proteins that are too big to be investigated with liquid-state NMR spectroscopy [1]. For example, Tycko and coworkers studied the folding of HP35 using frozen glycerol-water solution NMR in the presence of guanidine hydrochloride [2] or using fast freezing methods [3]. They also demonstrated the ability of frozen solution solid-state NMR to investigate large biomolecular complexes by studying HIV proteins bound to an antibody and RNA [4], [5]. Furthermore, frozen solution NMR of proteins and other biomolecules can be combined with dynamic nuclear polarization (DNP) a technique increasing the signal-to-noise (SN) of an NMR spectrum up to two orders of magnitude [6]. In fact, most of the biomolecular DNP done to date relies on frozen glycerol-water solutions, which serve as cryoprotectant and ensure the uniform distribution of the (bi)radicals [6].

However, solid-state NMR on frozen solutions comes at a price, since the linewidths observed for such samples, especially at the low temperatures of about 153 K used by Tycko and coworkers, and of about 100 K used for most DNP applications, are significantly larger than those observed in crystalline protein samples or other regular arrays of proteins [7]. We recently showed that it is possible to record relatively high resolution ^13^C spectra of ubiquitin and a type III antifreeze protein in frozen solution samples at higher temperatures, *i.e.* just a few degrees below the freezing point of bulk aqueous solvent [8].

These results raised in our minds the question of the detailed dependence of the linewidths on temperature and composition of the solvent between freezing and −80°C, where the hydration shell directly surrounding the protein is progressively becoming solid. Such a study could give some insights into the origin of the observed line broadening in frozen solutions.

Proteins in aqueous solution influence the properties of water in their direct vicinity. These water molecules, which form the hydration shell, reciprocally influence the structure and dynamic of the protein in solution [9]–[11]. Terahertz spectroscopy [12], neutron diffraction [13], and NMR [14]–[18] can be used to study the dynamics of the hydration shell and the interactions between the protein and its hydration water. Based on these methods and molecular dynamics simulations of protein-water system, it is believed that there is about 0.4 g of hydration water per gram protein and that this water is slightly denser and less dynamic than bulk water [14]. Several NMR studies suggested that a minimal protein hydration has not only a crucial influence on the dynamics of the protein, but also on the linewidth observed in solid-state NMR spectra of proteins, since the linewidth increases notably when the minimal hydration shell is removed [15], [19].

Interestingly, the hydration water of proteins does not freeze at the same temperature as the bulk solvent, but is mobile and non-crystalline over a considerable temperature range below the bulk transition temperature, and undergoes a glass transition at lower temperatures. The experimentally observed solvent glass transition temperature depends critically on the timescale of the method used to probe the hydration water [20]–[23], consistent with a continuous retardation of the translation timescale with temperature. For example, differential scanning calorimetry (DSC) data, probing macroscopic time scales (1–100 s), indicates a glass transition in frozen protein solutions of approximately 200 K [21]. Neutron diffraction and NMR data probe faster timescales and observe the glass transition at considerably higher temperatures. ^1^H NMR data indicate that the protein hydration shell of ubiquitin freezes and thereby escapes detection at about 223 K [24]. (This is confirmed in our NMR studies of the hydration shell of frozen ubiquitin and antifreeze protein solutions at 238 K [25]). Goddard and coworkers observed the complete arrest of the hydration water in rehydrated lyophilized protein at 170 K [18].

Since ^13^C NMR linewidths of proteins in frozen solution are often reported to be broad at temperatures below the protein-solvent glass transition, and relatively high-resolution ^13^C spectra of proteins could be obtained *above* this transition, we set out to answer the following questions: How does the ^13^C linewidth of a protein solution depend on temperature? What is the nature of the ^13^C line broadening? What influence do solvent dynamics and aggregation state have on the ^13^C linewidth? Is the ^13^C linewidth influenced by the addition of glycerol? To learn more about the relation between solvent dynamics and ^13^C linewidth, we present the following data on frozen ubiquitin solutions in the presence and absence of glycerol at temperatures down to 183 K.

## Results

### Carbon Linewidth

**Figure 1:**
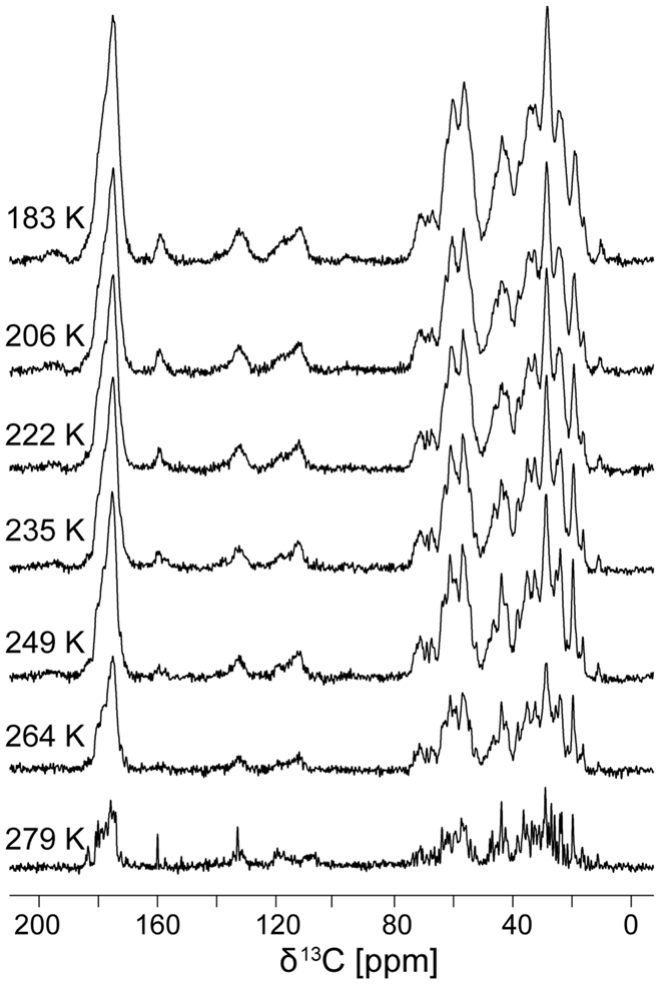
Temperature dependence of ^13^C linewidth in an ubiquitin-water solution. Spectra were recorded on a 750 MHz spectrometer, at 12 kHz MAS, using 128 acquisitions. Direct ^13^C excitation by a 90° pulse was used at 279 K, a ^1^H-^13^C CP pulse sequence was used otherwise. The spectra at 279 K and 264 K were recorded with less sample than the others explaining the reduced signal intensity. The spectra show that the ^13^C lines broaden abruptly when the solution freezes between 279 K and 264 K. After that, the linewidth increases in a continuous manner with lowering the temperature.

**Figure 2:**
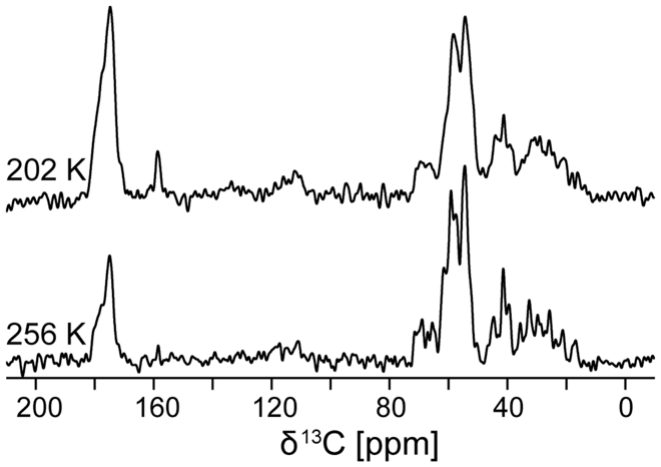
Temperature dependence of ^13^C linewidth of perdeuterated ubiquitin in frozen H_2_O solution. Spectra were recorded on a 750 MHz spectrometer, at 12 kHz MAS, using 1024 acquisitions. The increase in linewidth from 256 K to 202 K is similar to the line broadening observed on fully protonated ubiquitin. These data prove that imperfect decoupling is not the reason for the increase in linewidth.

How does temperature influence the ^13^C linewidth of a protein in H_2_O below the freezing point of the bulk solvent, *i.e.* trapped in ice? To answer this question, we recorded ^13^C spectra of ubiquitin at various temperatures. As can be seen from Figure 1, the linewidth of the spectra broadens in two stages. Noticeable broadening occurs with the freezing of the bulk water between 279 K and 264 K. As the temperature is further lowered from 264 K down to 183 K, the linewidth continuously broadens further. (To eliminate the possibility of a trivial effect on the decoupling performance, we re-optimized ^1^H decoupling at the lowest temperature, confirming the same decoupling optimum.) We recorded 1D CP MAS spectra on perdeuterated ubiquitin at 256 K and 202 K, and these samples exhibited linewidths very similar to those of protonated ubiquitin (see Figure 2). The observed line broadening was, therefore, not the result of insufficient decoupling.

**Figure 3:**
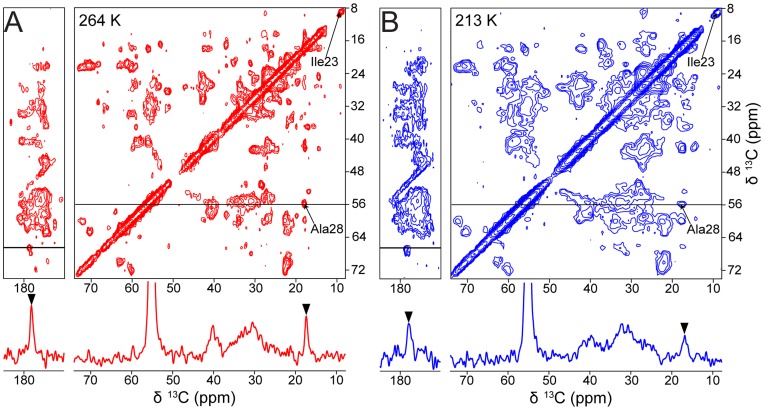
2D DARR spectra of U-^13^C-^15^N labeled ubiquitin in frozen H_2_O solution at 264 K and 213 K. A) Spectrum recorded at 264 K sample temperature (red). B) Spectrum recorded at 213 K sample temperature (blue). The 1D spectra shown are extracted form the δ_1_ slices indicated inside the 2D spectra. The two spectra illustrate the extent of line broadening induced by lowering the temperature in frozen protein solutions.

Figure 3 shows two 2D DARR ^13^C-^13^C correlation spectra we recorded at 264 K (red) and 213 K (blue) to monitor the extent of line broadening in more detail. The two spectra overlap very well giving little indication of chemical shift change between the two temperatures. The well-resolved Cδ1 of Il23 exhibits with about 0.5 ppm one of the highest chemical shift changes observed (see also reference [8]). However, the resolution at 213 K is significantly worse than at 264 K causing several cross peaks to merge at 213 K that were distinguishable at 264 K. The 1D slices shown below the 2D spectra confirm these results, and a dramatic line broadening could be observed *e.g.* for the Ala28 Cα-Cβ cross peak at 18.6 ppm.

The Cβ linewidth of this Ala28 increases from ∼150 Hz (0.8 ppm) at 264 K to ∼240 Hz (1.3 ppm) at 213 K. A similar extent of line broadening can be observed in Figure 2 where the isolated line of Ile23 Cδ1 at 8.5 ppm broadens from ∼100 Hz (0.5 ppm) at 279 K to ∼165 Hz (0.9 ppm) at 264 K and to ∼256 Hz (1.5 ppm) at 213 K.

**Figure 4:**
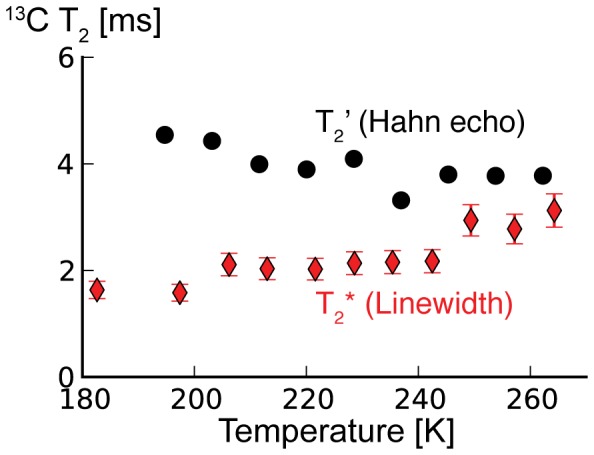
Temperature dependence of ^13^C T_2_’ of frozen ubiquitin-water solution in comparison with T_2_* calculated from the ^13^C linewidth. T_2_’ shown with black circles were measured with a simple Hahn echo pulse sequence and the data were fit to exponential decay curves. The values T_2_* plotted with red diamonds were calculated from the Cδ1 linewidth of Ile23. The T_2_’s measured with an echo sequence *increase* moderately towards lower temperatures and can, therefore, not explain the line broadening observed at lower temperatures.

**Figure 5:**
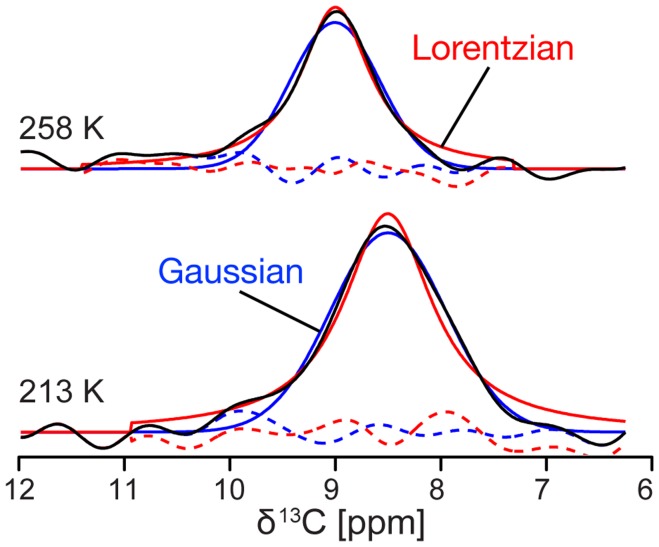
Change in ^13^C line shape of an ubiquitin-water solution with temperature. Fits of the Ile23 Cδ1 line of ubiquitin in frozen H_2_O solution to either Gaussian (blue) or Lorentzian (red) line shapes at two different temperatures. The residuals are shown with dashed lines in the same color. At 258 K the line can be fit better with a Lorentz function (χ^2^ = 5.7*10^14^) than with a Gaussian (χ^2^ = 1.9*10^15^) indicating a dominantly T_2_ relaxation based linewidth. At 213 K the line fits better to a Gauss (χ^2^ = 5.5*10^14^) than to a Lorentz (χ^2^ = 1.3*10^15^) function indicating that chemical shift distributions are more important for the linewidth at low temperatures. Also note the chemical shift change of ∼0.5 ppm.

Is this line broadening inhomogeneous, and due to an increase in structural heterogeneity at lower temperatures, or homogeneous, and due to an increase in T_2_ relaxation? To answer this question, we measured the temperature dependence of the average ^13^C T_2_’ of ubiquitin in frozen solution using a simple Hahn echo pulse sequence. Here, T_2_’ is the transverse relaxation time measured by a Hahn echo pulse sequence under MAS and high power ^1^H decoupling, which includes the spin-spin relaxation T_2_, the ^13^C-^13^C J-coupling, and contributions from imperfect averaging of dipolar couplings. The data in Figure 4 (circles) show that ^13^C T_2_’ *increases* with decreasing temperatures. In addition to this, Figure 4 shows the T_2_* calculated from the Cδ1 line of Ile23 (diamonds). T_2_* is the total transverse relaxation measured by the ^13^C FID under ^1^H decoupling, that includes T_2_’ and contributions from inhomogeneous line broadening *i.e.* small variations in the isotropic chemical shift throughout the sample. At temperatures just below the freezing point of the solution, the T_2_’ of ∼3.8 ms is only slightly longer than the T_2_* we calculated from the linewidth and most of the linewidth we observe is, therefore, due to T_2_’. However, the line broadening we observed at lower temperatures cannot be explained by T_2_’ alone. Line broadening and T_2_’ seem to be uncorrelated indicating that the line broadening we observe with lower temperatures is inhomogeneous. The fact that the Cδ1 line of Ile23 fit somewhat better to Lorentzian line shapes at high freezing temperatures and better to Gaussian line shapes at low temperatures, supports this finding (see Figure 5).

### Glycerol-water Solutions

**Figure 6:**
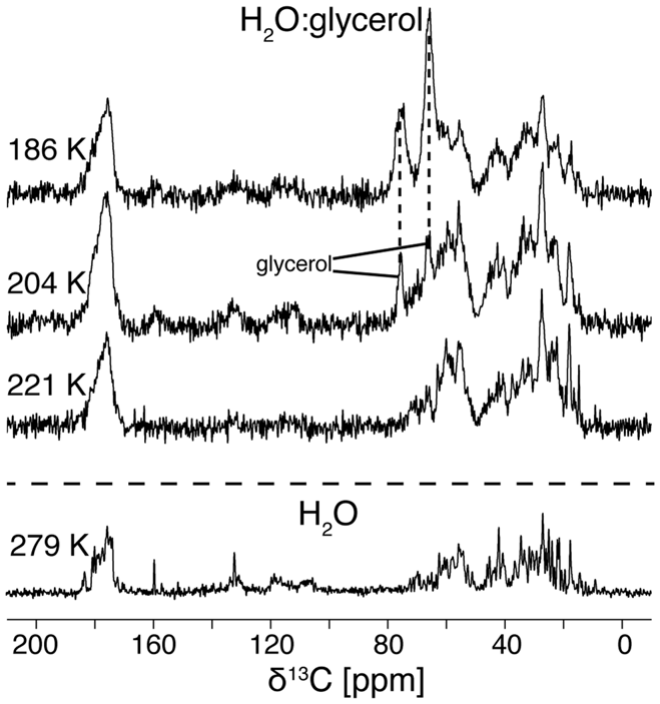
Temperature dependence of ^13^C linewidth of ubiquitin in 1∶1 v/v glycerol water solution. CP MAS spectra were recorded on a 750 MHz spectrometer, at 12 kHz MAS, using 128 acquisitions. Glycerol ^13^C lines are labeled with stars. As comparison, the equivalent spectrum of ubiquitin in H_2_O at 279 K from Figure 1 is also shown. The linewidth of the spectra increases when lowering the temperature similar to the spectra in non-glycerol solutions shown Figure 1. However, the line broadening occurs at lower temperatures compared to the solution without glycerol.

Many studies on frozen protein solutions are not done in frozen H_2_O, *i.e.* ice, but in mixtures of water and glycerol. In contrast to pure water, glycerol-water mixtures are known to form a glass at low temperatures, which proves to be advantageous, especially in the context of DNP experiments [6] and to prevent possible protein concentration and aggregation [26]. Therefore we asked: what is the influence of the additional glycerol on the ^13^C linewidth of ubiquitin in frozen solution? To answer these questions, we recorded spectra of ubiquitin in frozen 1∶1 glycerol-water mixtures. Figure 6 shows 1D ^13^C CP MAS spectra recorded between 221 K and 186 K. Above 221 K the sample turned out to be lossy and gave low CP efficiencies, indicating that it was still relatively liquid at these temperatures. Similar to the data recorded in pure water, the ^13^C linewidths of ubiquitin in glycerol-water are quite narrow right at the point where the viscosity of the bulk solvent slows the molecular tumbling of the protein into a range where the ^1^H-^13^C dipolar couplings are not averaged completely and the CP efficiency becomes comparable to crystalline samples. Lowering the temperature further leads to a continuous broadening of the protein linewidth. Furthermore, the glycerol becomes so rigid with lower temperatures that it is visible in the ^13^C CP spectra. The intensity of the ^13^C spectra we recorded of ubiquitin in glycerol-water solution is too low to observe the Ile23 line at 8.5 ppm, but already the three overlapping Cδs of Ile3, 13, and 61 at 13.9 ppm have a linewidth of less than 100 Hz (0.5 ppm) at 221 K. This linewidth corresponds to the linewidth of ubiquitin in H_2_O solution above freezing. When the temperature is lowered, these lines broaden considerably in both samples and overlap with other lines making linewidth measurements difficult.

### Magnitude and Dynamics of Non-frozen Water

**Figure 7:**
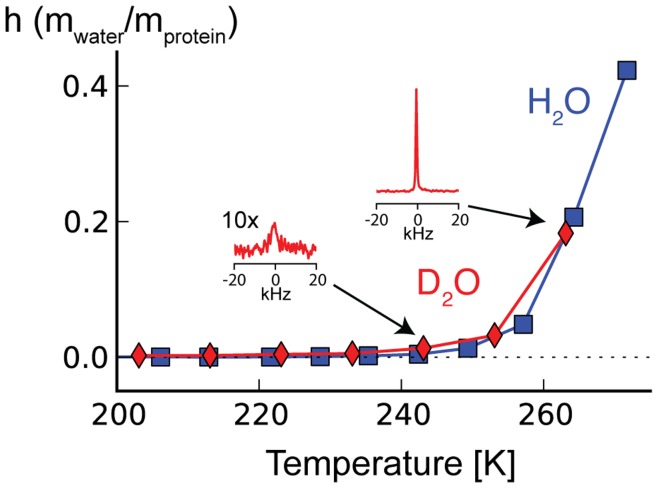
Temperature dependence of the ^1^H and ^2^H water line intensity in frozen ubiquitin solution. The ^1^H line intensity of the non-frozen water obtained by applying a sine bell square window function followed by magnitude mode processing (blue squares) becomes diminishing small around 240 K. The Intensity of non-frozen water ^2^H line of frozen ubiquitin-D_2_O solution (red diamonds) shows similar temperature dependence. Both ^1^H and ^2^H data were normalized to the respective signal intensity above the freezing point and expressed as g water per g protein. These data show that the non-frozen water becomes hard to detect in frozen ubiquitin solutions at about 240 K when observed with ^1^H and ^2^H NMR.

To learn more about how the ^13^C linewidth of ubiquitin in frozen solution is influenced by the dynamics of the surrounding water, we recorded ^1^H and ^2^H NMR spectra alongside the ^13^C spectra shown in Figure 1. Figure 7 shows the temperature dependence of the intensity of liquid-like ^1^H in a ubiquitin solution recorded at 750 MHz from temperature just below the freezing point down to about 200 K. The narrow ^1^H line of the non-frozen water was separated from broader, static ^1^H resonances using a sine bell window function and magnitude mode processing. The intensity of this narrow line reflects the amount of liquid water with a correlation time short enough to be detected in our experiment (much less than the inverse of the dipolar ^1^H-^1^H couplings, *i.e.* nanosecond or shorter). In these experiments, non-frozen water becomes undetectable at about 240 K. We recorded similar data for the non-frozen water in a frozen ubiquitin D_2_O solution using a static probe on a 600 MHz spectrometer (Figure 7). Also here we could observe a relatively narrow, sideband-free ^2^H line for the non-frozen water until it got less intense and difficult to detect at about 240 K. This narrow, sideband free ^2^H could not be detected in spectra of pure water and is also absent with some of the other solutes we tested. Only the broad ^2^H tensor of ice is detectable in this case.

**Figure 8:**
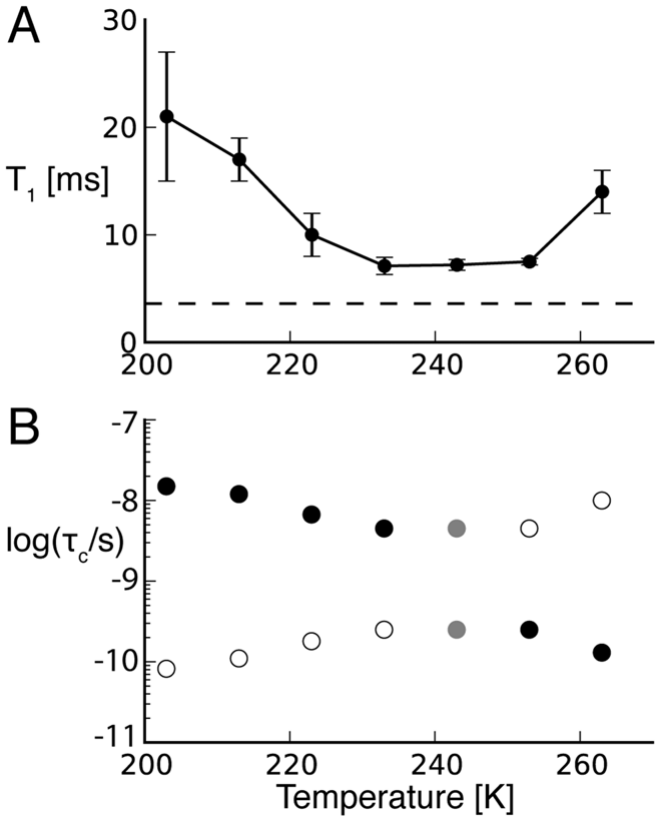
Water spin lattice relaxation time T_1_ of ^2^H of frozen ubiquitin solution and the resulting rotation correlation time τ_C_. A) ^2^H T_1_ of non-frozen water measured with a simple saturation recovery and quadrupolar echo pulse sequence. The theoretical minimum assuming a single τ_C_ and an isotopic motion is indicated with a dashed horizontal line. B) Temperature dependence of the non-frozen water τ_C_ calculated using Equation 1, the T_1_ relaxation times shown in A), and a quadrupolar coupling constant and asymmetry parameter of Cq = 195 kHz and η = 0.1 respectively. Two possible τ_C_ values correspond to each T_1_ in the temperature according to Equation 1. Since τ_C_ is expected to decrease at lower temperatures the most likely τ_C_s are shown with full circles. The equally likely τ_C_s corresponding to the minimal T_1_ are shown in gray.

To probe the ^1^H and ^2^H line broadening further, and clarify the slower molecular tumbling of the water at low temperatures, we measured ^2^H T_1_ and calculated the corresponding molecular correlations times τ_C_ of water using the following equation [27].
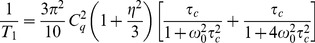
(1)C_q_ and η are the D_2_O quadrupolar constant and asymmetry parameter, respectively, and ω_0_ is the deuterium Larmor frequency. The ^2^H T_1_ of non-frozen water and the corresponding molecular correlations times assuming C_q_ = 195 kHz and η = 0.1 are shown in Figure 8. Despite the ambiguity of two solutions for the correlation time in this T_1_ measured, it is clear that that τ_C_ increases (to ∼15 ns) and the isotropic ^2^H lines disappears when the condition 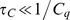 is not fulfilled [17]. The T_1_ relaxation times we measured did not reach the theoretical T_1_ minimum calculated from Equation 1 (dashed line in Figure 8a), which is often interpreted as evidence for complex motional models.

## Discussion

We reproduced an abrupt broadening of the ^13^C linewidth due to freezing of the solvent. Although this is not the major topic of the paper, it is interesting to discuss its origin. The abrupt broadening of the ^13^C linewidth of ubiquitin in H_2_O when freezing the bulk solvent between 279 K and 264 K might result from restriction of ubiquitin to the confined space of its hydration shell and therefore cessation of large-scale molecular motions that average the inhomogeneous environment. Similar effects could be expected due to excluded volume effects resulting from the formation of large ice crystals and a resulting concentration of protein [26]. Another possible explanation for broadening at the freezing point of the bulk solvent could be that the freezing introduces structural heterogeneity via direct ice interactions, *e.g.* the different orientations of crystal planes around the protein and resulting variations in the size and shape of the remaining hydration shell. These effects might explain the small difference at 263 K between the measured T_2_’ and the T_2_* calculated from the ^13^C linewidth (Figure 4).

Our major emphasis was on the behavior of the NMR linewidths as a function of temperature below the freezing point of the solution. We observed a gradual broadening of the ^13^C line when the temperature was lowered below the freezing point down to 183 K. It is interesting to compare this temperature profile to that of the solvent motions: The ^2^H and ^1^H lines of the non-frozen water undergo a more marked broadening and loss of detectability at about 240 K. Over this narrower temperature range, 240–260 K, where the solvent apparently undergoes a transition, we did not observe a more substantial ^13^C linewidth change; the linewidth change for the protein was relatively smooth over a much broader temperature range. This observation suggests that there is no specific relation between solvent dynamics or averaging and linewidth over this range.

The additional ^13^C line broadening at 183 K as compared with 264 K is inhomogeneous. T_2_’ of the protein ^13^C lines *increases* with lower temperatures over this range. The line broadening does not result from a decrease in T_2_ but from an increase in local inhomogeneity. At high temperatures, it is possible that fast exchange among local microstates leads to narrow ^13^C lines. At lower temperatures, the local conformations appear to become increasingly locked, leading to conformational differences throughout the sample, that are distinct and long-lived on the NMR timescale (ms or slower), and distinct in terms of their isotropic chemical shift, and, therefore, line broadening is observed. This also explains why the line shapes become less Lorentzian and increasingly Gaussian at lower temperatures: Just below the freezing point of the solution the linewidth comes dominantly from the exponential T_2_’ decay of the ^13^C magnetization resulting in a Lorentzian line shape. At low temperatures the linewidth comes increasingly from a distribution of chemical shifts resulting in a Gaussian line shape. The linewidth change can be thought of as local, relatively modest structural heterogeneity. The difference in linewidth of Ala28’s Cβ between 264 K and 213 K is ∼0.5 ppm. In comparison, the average difference between an Ala Cβ in an α-helical and extended β-sheet conformation is about 3.5 ppm. The line broadening of 0.5 ppm corresponds therefore presumably to a change that is much less dramatic than a secondary structure change. The line broadening at low temperatures is not typically accompanied with a measurable change in average chemical shift; only a few isolated signals such as the Cδ1 of Ile23 showed chemical shift changes in the order of 0.5 ppm. Interestingly, Ile23 also showed the maximum chemical shift in an earlier study comparing solution with frozen solution shifts [8], suggesting that this is an unusually plastic site in the protein.

Therefore, the ^13^C line broadening does not reflect a change of secondary and tertiary structure of the protein but can be explained by continuous arrest of rotational transitions of amino acid side chains that are lubricated by the presence of a solvent shell or, especially in the case of methyl rotations, are volume conserving and independent of solvent dynamics [28], [29].

Maus and co-workers described ^13^C line broadening at low temperatures due to the interference between the methyl group rotation and ^1^H decoupling field strength in a crystalline sample [30]. The fact that we observed comparable line broadening in protonated and perdeuterated samples of ubiquitin rules out the possibility that insufficient ^1^H decoupling or interference between the methyl group rotation and ^1^H decoupling field strength could be the reason for the line broadening in our case. However, the freezing of methyl group rotation could play an important role for the ^13^C linewidth at lower temperatures [31].

The glycerol-water solution has no liquid-solid phase transition and rigidifies at much lower temperatures compared to pure water. Therefore, ubiquitin in glycerol-water becomes accessible to dipolar CP MAS spectroscopy at lower temperatures than ubiquitin in pure water. The ^13^C lines of the protein in glycerol water mixtures become gradually broader as the temperature is lowered, analogously to the protein frozen in ice. However, at a given temperature the protein lines in glycerol-water mixtures are considerably narrower. We observed ^13^C linewidths at 221 K in glycerol-water that are comparable to those in pure water at 264 K [32]. Between the freezing point of the pure water and the glass transition of the glycerol-water solution (∼160 K), the bulk glycerol-water solution is apparently more dynamic than pure water at this temperature. It is very likely that this is also true for the water in the vicinity of the protein, leading to narrower lines at comparable temperatures. However, we were not able to distinguish the solvent at the protein surface from the bulk solvent with NMR in the case of the glycerol-water mixture.

It is curious that for glycerol-water solutions over the temperature range from 221 to 186 K, dipolar based NMR sequences that would normally require a solid sample, such as CP, perform well, despite the fact that the solvent has not undergone a glass transition. This is arguably analogous to the situation for crystalline protein samples that are typically bathed in a liquid medium between about 200 and 270 K, and give very high quality SSNMR spectra nevertheless, because they are arrested by protein-protein interactions. Mainz and coworkers previously observed high-resolution ^13^C CP MAS spectra of a protein in 20% glycerol-water solutions at a sample temperature of 263 K; the ability to achieve a high quality solid-state NMR signal at this relatively high temperature was due to sedimentation of the protein complex, rather than a rotational correlation time slow enough to permit Hartman-Hahn cross polarization [33]. For ubiquitin the centrifugal forces of an MAS rotor are not sufficient to sediment the protein. Certainly a precipitate of the protein could be centrifuged but an arrest of the protein due to the solidification of the solvent is the more likely reason that dipolar based solid-state NMR techniques are applicable to our samples [33].

The temperature at which NMR of frozen solutions show the best resolution, in pure water and glycerol-water mixtures, is as close as possible to the bulk transition, or the point where the solution is rigid enough to allow dipolar CP to work. At lower temperatures the line broadening is sufficient to present practical problems for example by making the assignment of 2Ds (Figure 3) increasingly difficult. In this case selectively labeling or additional spectral dimensions become necessary [2], [34]. The linewidths we observed in frozen ubiquitin solutions at all temperatures are well above those observed by Zech and coworkers in crystalline samples of ubiquitin under very similar conditions [35]. This difference may result from larger structural inhomogeneity in the frozen than in the crystalline state. It is worth noting, however, that the linewidth of crystalline proteins can also get larger when lowering the temperature as characterized in a recent study about the temperature dependence of the ^13^C linewidth in crystalline SH3 [36]. Rapid freezing experiments have been proposed to obviate this problem, but Hu and coworkers showed that the speed of freezing has only a modest effect on the linewidth of natively folded protein in frozen solution [3] and a recent EPR study showed that the freezing speed has no effect on protein structure [26]. Thus, other solutions are needed to this important practical problem.

We were able to observe a significant fraction of mobile water in ubiquitin in H_2_O below the freezing point of the bulk water. When expressed in a mass ratio of water per protein, we find that this ratio corresponds relatively well to the 0.4 g of hydration water per g of protein reported in the literature [14]. In a previous publication we measured ^1^H -^13^C correlation spectra that showed that this water is in exchange with the protein [25]. We therefore assign this non-frozen water to either the direct hydration shell of the protein or water that is in rapid exchange with the hydration shell.

We observed that the intensity of the ^1^H and ^2^H lines of the non-frozen water in Figure 7 are much diminished at 240 K (as compared to 270 K). In contrast, Tompa and coworkers reported relatively constant ^1^H line intensity between 273 and 228 K by analyzing the components of a static ^1^H spectrum (wide-line NMR) of a very similar frozen ubiquitin solution. Interestingly, our intensity measurements correspond very well to the DSC data reported in the same study [24]. The rotation correlation time τ_C_ of the non-frozen water measured via the ^2^H T_1_ relaxation rate became longer when lowering the temperature of our frozen protein solution (see Figure 8a). Furthermore, the work of Vogel and coworkers indicated that these dynamics are also complex [17]. This continuous change in complex solvent dynamics underlies the glass transition, whose temperature depends partly on the measurement criterion or the timescale of the experimental method. Our observation of a strong broadening of the ^1^H and ^2^H non-frozen water lines around 240 K, suggests that at this temperature τ_C_ was in the order of 10^−10^–10^−8^ s and the 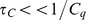 condition was not fulfilled any more. This timescale for water rotation corresponds very well to the correlation times reported by Vogel and coworkers, who used ^2^H spectra and T_1_ measurements to characterize the dynamics of supercooled water in wet protein samples. The T_1_ data reported in that study fit best to a distribution of correlation times. Such a distribution of correlation times could also explain the discrepancy between the theoretical T_1_ minimum and the minimal measured T_1_ in our data (see Figure 8). Additional data at different magnetic fields would be needed to characterize this distribution in more detail.

The ^13^C linewidth of the protein behaves very differently from the ^1^H and ^2^H linewidth of the non-frozen water. The narrow lines at high temperatures are assumed to be the result of dynamic averaging over different conformations in the fast exchange limit. The broad lines at lower temperatures are the result of the reduction of these dynamics, freezing of multiple specific conformations, which leads to static disorder and inhomogeneous line broadening. The timescale and of the motions relevant for the ^13^C linewidth are determined by the intermediate to slow exchange limit given by the heterogeneous ^13^C chemicals shifts. Assuming a line broadening of about 100 Hz, the motions leading to line narrowing at high temperatures are expected to be much faster than 10^−3 ^s, and at lower temperatures must be much slower than that timescale. The ^13^C linewidth is, therefore, potentially sensitive to much slower motions than the ^1^H and the static ^2^H lines.

From these data, the linewidth changes that we observe between 190 and 270 K are not likely due to the changes in solvent dynamics we observe via the ^1^H and ^2^H spectra of the non-frozen water. However, the fact that the ^13^C line broadening we observe is continuous with temperature does not exclude the possibility that the change in linewidth is connected to other dynamical modes or timescales of the surrounding solvent. Bajaj and coworkers observed no significant ^13^C line broadening in spectra of a dehydrated crystalline tripeptide down to temperatures of 90 K [37]. Linden and coworkers, on the other hand, observed very similar line ^13^C line broadening in SH3 protein crystals that contain significant amounts of crystal water [36]. Side-chain rotamer motions, especially methyl rotations that could be responsible for the ^13^C line broadening we observe have been implicated to be solvent independent [28]. On the other hand the motions of especially solvent accessible polar sidechains are thought to be facilitated by the presence of the solvent and arrest if the solvent shell is either absent or very viscous [18]. Dry protein samples are usually showing severe ^13^C line broadening which disappears with the hydration of the protein [15], [19]. This indicates that water plays a role for the slow dynamics governing the ^13^C linewidth and has a function as lubricant of protein motions.

The fact that ubiquitin does not make direct contact to the surrounding ice but rather has a glassy hydration shell at all temperatures [21] and the fact that non-ice forming glycerol solutions show similar line broadening effects than pure H_2_O solutions make it unlikely that the ^13^C line broadening we observed is a result of sample heterogeneity due to spontaneous formation of crystalline ice at the protein surface.

This model could be further tested in a number of ways. Extending the data presented in this manuscript to temperatures below 180 K would be desirable since they would answer the question whether the broadening of the ^13^C lines continues at lower temperatures similar to crystalline proteins [36] or reaches a plateau when the timescale of the side-chain motions become of the order of milliseconds. Another interesting way of testing the abovementioned hypothesis is the measurement of dynamic order parameters via *e.g.* ^1^H-^13^C dipolar couplings that could identify the difference in dynamics of the protein in frozen solution above the protein-solvent glass transition and at very low temperatures.

In summary, we have characterized the ^13^C linewidth of ubiquitin and its dependence on non-frozen solvent dynamics in frozen pure H_2_O and glycerol-water solutions at different temperatures. In pure H_2_O solutions, after an abrupt line broadening due to the freezing of the bulk solvent, the protein ^13^C linewidth broadens continuously when lowering the temperature. This line broadening is inhomogeneous and independent of ^1^H decoupling as confirmed with Hahn echo experiments and perdeuterated samples, respectively. The ^13^C linewidth of a protein in frozen solution does not follow the pronounced line broadening we observed of the hydration shell ^1^H and ^2^H line around 240 K. We hypothesize that the ^13^C line broadening may be related to solvent motions other than those we observed using ^1^H and ^2^H NMR. In this picture, the protein ^13^C linewidth depends first on the freezing point of the solvent, arresting the protein to a confined space and, after the bulk solvent is frozen, on the local dynamics of the protein and its hydration shell. The glycerol-water mixture does not undergo a sharp phase transition and we consequently did not observe an abrupt ^13^C line broadening as in pure H_2_O. Generally, the ^13^C linewidth in glycerol-water was smaller than in pure H_2_O at the same temperature but the linewidth increased when lowering the temperature analogous to the pure H_2_O solution.

## Materials and Methods

### Sample Preparation

Uniformly ^13^C-^15^N labeled ubiquitin was expressed and purified as described earlier [25]. The lyophilized protein was dissolved in deionized water and 1∶1 v/v glycerol-water at final concentrations of 35 mg/ml and 32 mg/ml, respectively.

### NMR

Solid-state ^13^C and ^1^H NMR spectra were recorded on a Bruker 750 MHz Advance spectrometer using a 4 mm double resonance probe operating at 12 kHz MAS. The sample temperatures were calibrated externally using Pb(NO_3_)_2_ [38]. ^1^H 1D spectra and saturation recovery curves were recorded using hard pulses of 100 kHz. Protein 1D ^13^C spectra in frozen solutions were recorded using ^1^H-^13^C CP experiments with a contact time of 1 ms and rf-field strengths of 50 and 62 kHz for ^13^C and ^1^H, respectively. Equivalent ^13^C 1D spectra of ubiquitin solutions above the freezing point were recorded using direct excitation via a 90° pulse of 50 kHz. Spinal64 [39] decoupling with a ^1^H rf-field strength of 100 kHz was used during all ^13^C detection periods. The ^13^C T_2_’ relaxation of ubiquitin and glycerol were measured with CP step followed by a simple ^13^C Hahn echo element and detection on ^13^C.

The 2D DARR spectra of Figure 3 were recorded with a mixing time of 20 ms. The spectral width was 50 kHz in both dimensions and 1536 and 1024 points were acquired in the t_1_ and t_2_ dimensions, respectively. For each t_1_ increment 16 and 64 scans were acquired for spectra at 213 K and 264 K, respectively.

Deuterium spectra were recorded on a Varian 600 MHz InfinityPlus spectrometer using a static ^2^H probe. T_1_ was measured by saturation-recovery followed by a quadrupolar echo with t = 23 µs. The T_1_ of non-frozen water and of ice differ by several orders of magnitude making it is easy to separate the spectra of each component.
